# Distilling structure in Taverna scientific workflows: a refactoring approach

**DOI:** 10.1186/1471-2105-15-S1-S12

**Published:** 2014-01-10

**Authors:** Sarah Cohen-Boulakia, Jiuqiang Chen, Paolo Missier, Carole Goble, Alan R Williams, Christine Froidevaux

**Affiliations:** 1 Laboratoire de Recherche en Informatique, CNRS UMR 8623, Université Paris Sud, France; 2 AMIB group, INRIA Saclay, France; 3 School of Information Science and Engineering, Lanzhou University, Lanzhou, Gansu, China; 4 University of Newcastle, UK; 5 University of Manchester, UK

## Abstract

**Background:**

Scientific workflows management systems are increasingly used to specify and manage bioinformatics experiments. Their programming model appeals to bioinformaticians, who can use them to easily specify complex data processing pipelines. Such a model is underpinned by a graph structure, where nodes represent bioinformatics tasks and links represent the dataflow. The complexity of such graph structures is increasing over time, with possible impacts on scientific workflows reuse. In this work, we propose effective methods for workflow design, with a focus on the Taverna model. We argue that one of the contributing factors for the difficulties in reuse is the presence of "anti-patterns", a term broadly used in program design, to indicate the use of idiomatic forms that lead to over-complicated design. The main contribution of this work is a method for automatically detecting such anti-patterns, and replacing them with different patterns which result in a reduction in the workflow's overall structural complexity. Rewriting workflows in this way will be beneficial both in terms of user experience (easier design and maintenance), and in terms of operational efficiency (easier to manage, and sometimes to exploit the latent parallelism amongst the tasks).

**Results:**

We have conducted a thorough study of the workflows structures available in Taverna, with the aim of finding out workflow fragments whose structure could be made simpler without altering the workflow semantics. We provide four contributions. Firstly, we identify a set of anti-patterns that contribute to the structural workflow complexity. Secondly, we design a series of refactoring transformations to replace each anti-pattern by a new semantically-equivalent pattern with less redundancy and simplified structure. Thirdly, we introduce a distilling algorithm that takes in a workflow and produces a distilled semantically-equivalent workflow. Lastly, we provide an implementation of our refactoring approach that we evaluate on both the public Taverna workflows and on a private collection of workflows from the BioVel project.

**Conclusion:**

We have designed and implemented an approach to improving workflow structure by way of rewriting preserving workflow semantics. Future work includes considering our refactoring approach during the phase of workflow design and proposing guidelines for designing distilled workflows.

## Background

Scientific workflows management systems [1-5] are increasingly used to specify and manage bioinformatics experiments. Their simple programming model appeals to bioinformaticians, who can use them to easily specify complex data processing pipelines. However, as stated by recent studies [6-8], while the number of available scientific workflows is increasing along with their popularity, workflows are not (re)used and shared as much as they could be.

In this work, we have focused specifically on the Taverna workflow management system [9], which for the past ten years has been popular within the bioinformatics community [1]. Despite the fact that hundreds of Taverna workflows have been available for years through the myExperiment public workflow repository [10], their reuse by scientists other than the original author is generally limited. Some of the causes for the limited reuse have been identified in the sheer difficulty to preserve a workflow's functionality vis-a-vis the evolution of the services it depends on [11]. In addition to this, another factor that limits reuse is the complexity of workflow structure, that involves the number of nodes and links but is also related to intricate workflow structure features. Several factors may explain such a structural complexity including the fact that the bioinformatics process to be implemented is intrinsically complex, or the workflow system may not provide appropriate expressivity, forcing users to design arbitrary complex workflows.

In the present work, the system considered is Taverna. Our approach aims at automatically detecting parts of the workflow structure which can be simplified by removing explicit redundancy and proposing a possible workflow rewriting. Our preliminary analysis of the structure of 1,400 scientific workflows collected from myExperiments reveals that, in numerous cases, such a complexity is due mainly to redundancy, which is in turn an indication of over-complicated design, and thus there is a chance for a reduction in complexity which does not alter the workflow semantics. Our main contention in this paper is that such a reduction in complexity can be performed automatically, and that it will be beneficial both in terms of user experience (easier design and maintenance), and in terms of operational efficiency (easier to manage, and sometimes to exploit the latent parallelism amongst the tasks).

Our specific contribution is a method for the automated detection and correction of certain Taverna workflow structures which can benefit from refactoring. We call these idiomatic structures 'anti-patterns', that is, patterns that should be avoided. Our approach involves the detection of several anti-patterns and the rewriting of the offending graph fragment using a new pattern that exhibits less redundancy and simpler structure while preserving the semantics of the original workflow. We have then designed the *DistillFlow *algorithm and evaluated its effectiveness both on a public collection of Taverna workflows and on a private collection of workflows from the BioVel project.

The rest of the paper is organized as follows. The Background section will continue by briefly summarize the Taverna workflow system features. In the Methods section we will introduce the anti-patterns we have identified and the transformations we propose to do while ensuring that the semantics of the workflow remains unchanged. We will then introduce the DistillFlow refactoring algorithm. In the Results section, we provide the results obtained by our approach on a large set of real workflows.

### Workflows in Taverna

As mentioned earlier, this work is specific to the Taverna workflow model [1], which we briefly summarize here. Examples of Taverna workflows are given throughout the paper. Taverna combines a dataflow model of computation with a functional model that accounts for list data processing. A workflow consists of a set of processors, which represent software components such as Web Services and may be connected to one another through data dependencies links. This can be viewed as a directed acyclic graph in which the nodes are processors, and the links specify the data flow. Processors have named input and output ports, and each link connects one output port of a processor to one input port of another processor. A workflow has itself a set of input and output ports, and thus it can be viewed as a processor within another workflow, leading to structural recursion.

**Figure 1:**
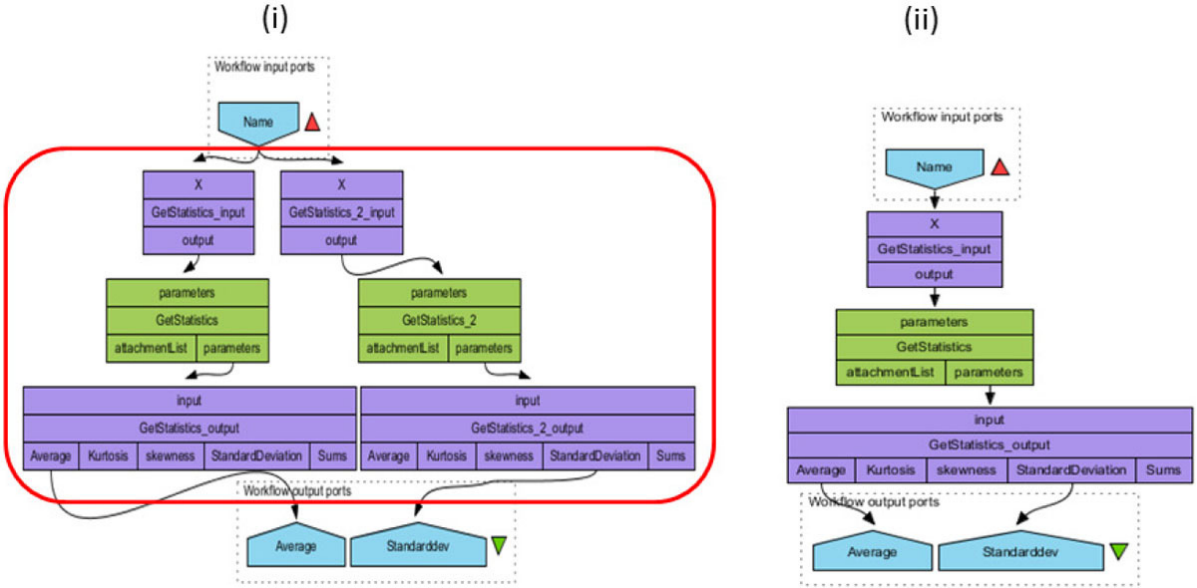
**Example of workflow (myExperiment 2383)**. Example of a Taverna workflow extracted from myExperiment. On the left hand side (numbered (i)) the original workflow is displayed and a red box highlights the part where redundancies occur. The workflow depicted on the right hand side of the figure (numbered (ii)) is a semantically-equivalent workflow with no redundancies.

The workflow depicted in Figure 1 (i), for instance, has one input called *Name *and two outputs named respectively *Average *and *Standarddev*. In turn, processor *GetStatistics_output *has one input port named *input *and five output ports named *Average*, *Kurtosis*, *Skewness*, *StandardDeviation *and *Sums*. We call the triple 〈< workflow name >, < workflow inputs >, < workflow outputs >〉 the *signature *of the workflow.

Note that multiple outgoing links from processors or inputs are allowed, as is the case for the workflow input of Figure 1 (i) which is used by two processors. Also, not all output ports must be connected to downstream processors (e.g., the value on output port *attachment_list *in *Get_Statistics *is not sent anywhere), and symmetrically, not all inputs are required to receive an input data (but input ports with no incoming links should have a default value, or else the processor will not be activated).

Input ports are statically typed, according to a simple type system that includes just atomic types (strings, numbers, etc.) and lists, possibly recursively nested (i.e., the type of a list element may be a list, with the constraint that all sub-lists must have the same depth). The functional aspects of Taverna come into play when one or more list-value inputs are bound to processor's ports which have an atomic type (or, more generally, whose nesting level is less than the nesting level of the input value). In order to reconcile this mismatch in list depth, Taverna automatically applies a higher-order function, the *cross product*, to the inputs. The workflow designer may specify an alternative behavior by using a *dot product *operator instead. This produces a sequence of input tuples, each consisting of values that match the expected type of their input port. The processor is then activated on each tuple in the list. There resulting "implicit iteration" effect can be defined formally in terms of recursive application of the *map *operator [12].

## Methods

This section begins by illustrating the two main types of anti-patterns found by our workflow survey, by means of two use cases. The formalization of the anti-patterns and the *DistillFlow *algorithm will be then introduced.

### Use cases

The first use case (Figure 1 (i)) involves the duplication of a linear chain of connected processors *GetStatistics_input*, *GetStatistics *and *GetStatistics_output*. The last processor in the chain reveals the rationale for this design, namely to use *one output port from each copy of the processor*. Clearly, this is unnecessary, and the version in Figure 1 (ii) achieves the same effect much more economically, by drawing both output values from the same copy of the processor.

**Figure 2:**
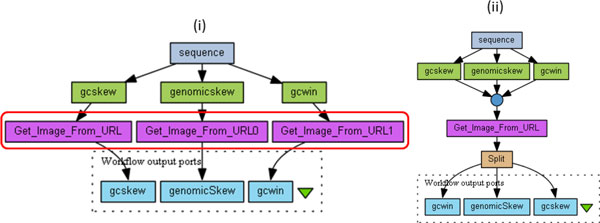
**Example of workflow (myExperiment 778)**. Example of a Taverna workflow extracted from myExperiment. On the left hand side (numbered (i)) the original workflow is displayed and a red box highlights the part where redundancies occur. The workflow depicted on the right hand side of the figure (numbered (ii)) is a semantically-equivalent workflow with no redundancies. A *merge *node (circle) and a *split *node have been introduced.

In the second use case (Figure 2 (i)), the workflow begins with three distinct processing steps on the same input sequence. We observe that the three steps that follow those are really all copies of a master *Get_image_From_URL *task. This suggests that their three inputs can be collected into a list, and the three occurrences can be factored into a single occurrence which consumes the list. By virtue of the Taverna list processing feature described earlier, the single occurrence will be activated three times, one for each element in the input list. Also, the outputs of the repeated calls of *Get_image_From_URL *will be in the same order as items in the list. Therefore this new pattern achieves the same result as the original workflow. Note that collecting the three outputs into a list requires a new built-in *merge *node (the circle icon in Figure 2 (ii)). Similarly, a *Split *processor has been introduced to decompose the outputs (list of values) into three single outputs.

**Figure 3:**
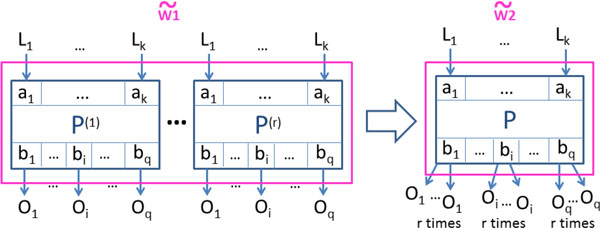
**Transformation for anti-pattern (A)**. Anti-pattern (A) and a semantically-equivalent transformation produced by our approach (to go from $\tilde{w}\text{1}$ to $\tilde{w}\text{2}$).

**Figure 4:**
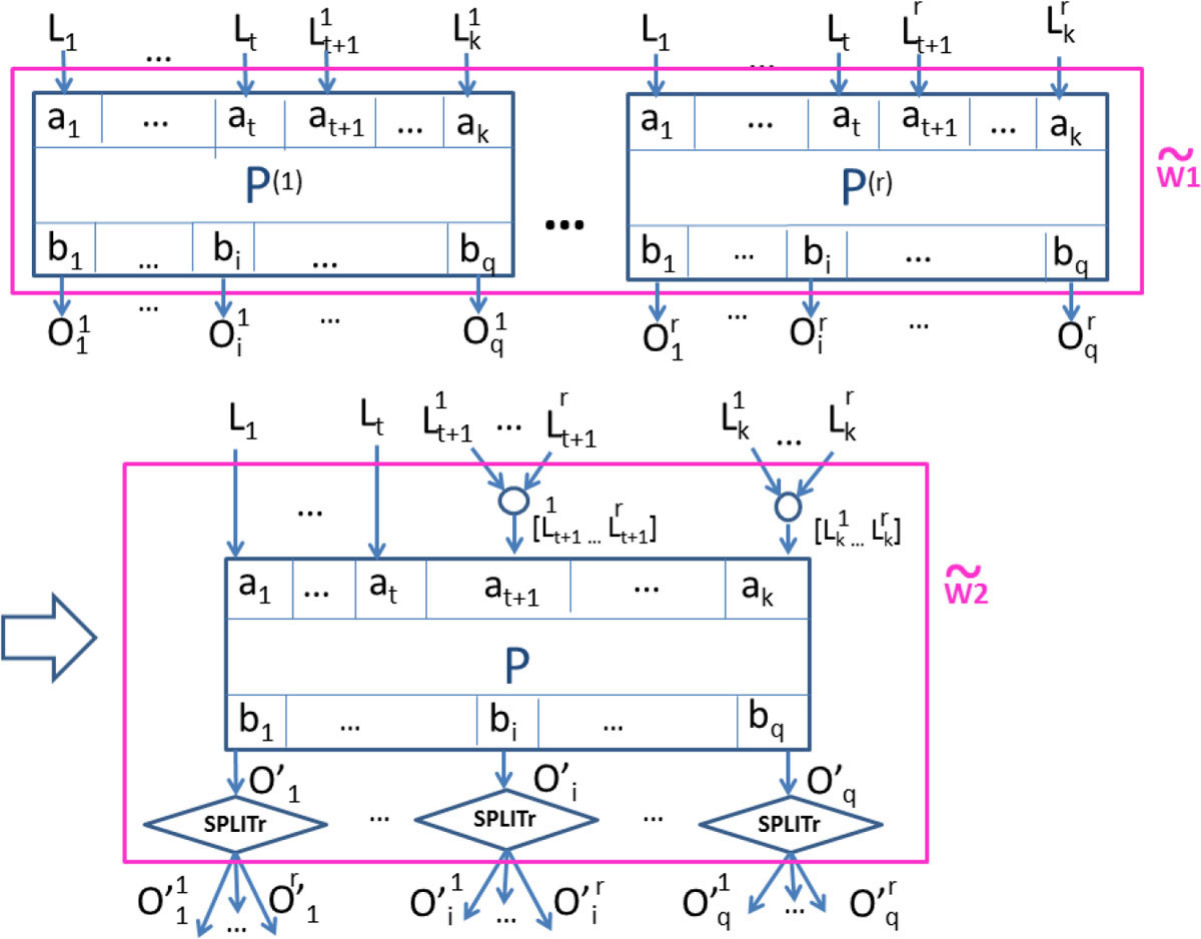
**Transformation for anti-pattern (B)**. Anti-pattern (B) and a semantically-equivalent transformation produced by our approach (to go from $\tilde{w}\text{1}$ to $\tilde{w}\text{2}$).

These two examples are instances of the general patterns depicted in Figures 3 and 4 (left hand side). These are the *anti-patterns *we alluded to earlier, and our goal is to rewrite them into the new structures shown in the right hand side of the figures. In the rest of this section we describe this rewriting process in detail.

### Anti-patterns and transformations

The transformations aim at reducing the complexity of the workflow by replacing several occurrences of the same processor with one single occurrence whenever possible. Although new processors are sometimes introduced in the process (i.e., merge and split operators), on balance we expect a cleaner design, better use of the functional features of Taverna (automated list processing) and lower redundancy, and thus fewer maintenance problems.

#### Assumptions

The following four assumptions must hold for processor instances to be candidates for the transformations described below.

1. A processor must be **deterministic**: it should always produce the same output given the same input.

2. Only processors implemented using the **exact same code **can be merged. Determining that two processors are equivalent is an open problem (see e.g. [6] for a discussion on that point) since it is directly associated to determining the equivalence of programs. In our setting, two processors are equivalent if they represent identical web service calls, or they contain the same script, or they are bound to the same executable Java program. In practice, this condition is often realized, because processors are duplicated during workflow design by means of a graphical "copy and paste" operation.

3. Only copies of processors that **do not depend on each other **can be merged, that is, if *P*^(1) ^and *P*^(2) ^are two occurrences of the same processor *P*, then there should not be any directed path between *P*^(1) ^and *P*^(2)^, for *P*^(1) ^and *P*^(2) ^to be merged.

4. We will consider only two cases where we can be sure that the **same input va**l**ue ***L_i _*can be bound to the input port *a_i _*of *r *copies of *P*: (a) the input port *a_i _*is bound to a constant value which is identical across executions (that is, among different copies) of *P*, or (b) *L_i _*has been produced by the output port of some processor *Q_i _*and has been distributed to the *r *copies of *P*.

#### Transformations

The two proposed transformations are shown in Figures 3 and 4, where each *P*^(*l*) ^(1 **≤ ***l ***≤ ***r*) denotes an occurrence (i.e., a copy) of processor *P*, with input and output ports *a*_1_, ..., *a_k _*and *b*_1_, ..., *b_q_*, respectively.

**Anti-pattern A: **In the first anti-pattern (Figure 3), the input ports *a_i _*of each processor occurrence *P*^(*l*) ^are all bound to the same value *L_i_*, for 1 **≤ ***i ***≤ ***k*, 1 **≤ ***l ***≤ ***r*. It follows from our assumption of determinism that the output ports *b_j _*all present the same output value *O_j _*across all *P*^(*l*)^, for 1 **≤ ***j ***≤ ***q*.

The rewriting replaces all *P*^(*l*) ^with a single occurrence, *P*.

***Treatment of the outputs: ***Outgoing links are then added to ports *b_j _*as needed.

***Treatment of the inputs: ***For each input port *a_i _*of *P*, the unique input value *L_i _*bound to *a_i _*is now either the constant value as previously in the (original) anti-pattern (cf. assumption 4.(a)), or it is one of the distributed values bound to some output port of some processor *Q_i _*(assumption 4.(b)) and in this last case processor *Q_i _*does not need to distribute this output value more than once anymore.

**Illustration: **One example of anti-pattern A is depicted on Figure 1 (i) where the same workflow input is sent to two exact copies of the processor *GetStatistics_input*. The workflow input plays the role of processor *Q*. *GetStatistics_input *and *GetStatistics_*2*_input *are thus merged and the workflow input (*Name*) is sent only once to the downstream of the workflow, that is, to the (now) single *GetStatistics_input *processor. Outputs are linked to the rest of the workflow and transformations must be applied as many times as necessary. In this example, three successive transformations are applied thus giving the workflow of Figure 1 (ii).

**Anti-pattern B: **In the second pattern (Figure 4), the input ports *a_i _*of each processor occurrence *P*^(*l*) ^are bound to the same value *L_i_*, for 1 **≤ ***i ***≤ ***t *while the input ports *a*_*t *+ 1 _to *a_k _*of each processor occurrence *P*^(*l*) ^are bound to different inputs $L_{t+1}^{l}$ to $L_{k}^{l}$ among occurrences, 1 **≤ ***l ***≤ ***r*. As for output values, let $O_{i}^{l}=P^{\left(l\right)}{|}_{bi}\left(L_{\text{1}},.\;.\;.\;,L_{t},L_{t+1}^{l},.\;.\;.\;,L_{k}^{l}\right)$ denotes the output value produced by output port *b_i _*of the *l*-th occurrence of *P*. For the sake of generality, we consider here that processor *P *applies cross product to values on ports *a*_1 _to *a_t _*and dot product to values on ports *a*_*t *+ 1 _through *a_k_*.

The rewriting replaces all *P*^(*l*) ^with a single occurrence, *P*.

Input data that differ from one occurrence to another ($L_{t+1}^{l}$ to $L_{k}^{l}$) have been merged using the merge processors provided by Taverna (the circle icon in Figure 4) to construct lists of data from the original data items to exploit the implicit iterative process of Taverna. As a consequence, the outputs of *P *are lists of data instead of single values in the original pattern. Since *P *follows a dot strategy on ports *a*_*t *+ 1_... *a*_*k*_, $O^{\prime }{\;}_{i}$ is the list $\begin{array}{l} O^{\prime }{\;}_{i}=[P{|}_{bi}(L_{\text{1}},\ldots ,L_{t},L_{t\text{+1}}^{1},\ldots ,L_{k}^{1}),\ldots ,\\ \;P{|}_{bi}(L_{\text{1}},\ldots ,L_{t},L_{t\text{+1}}^{l},\ldots ,L_{k}^{l}),\ldots ,P{|}_{bi}(L_{\text{1}},\ldots ,L_{t},L_{t\text{+1}}^{r},\ldots ,L_{k}^{r})] \end{array}$, for output port *b_i_*, 1 ≤ *i *≤ *q*.

***Treatment of the outputs: ***For each output port *b_i _*of *P*, the rewritten pattern contains a *list split *processor called *SPLIT_r _*to decompose the list obtained into *r *pieces so that the downstream fragment of the workflow remains unchanged. We get: $O\prime_{i}^{l}=P{|}_{bi}\left(L_{1},\ldots ,L_{t},L_{t+1}^{l},\ldots ,L_{k}^{l}\right)\left(1\leq l\leq r\right)$.

***Treatment of the inputs: ***Note that for each input port *a*_*t*+1_,..., *a_k_*, input values $L_{i}^{l}$ are used in the same way both before and after the transformation (1 **≤ ***l ***≤ ***r*, *t *+ 1 **≤ ***i ***≤ ***k*). As for input ports *a*_1 _to *a_t_*, instead of having *r *occurrences, each *L_i _*has now one single occurrence, 1 **≤ ***i ***≤ ***t *(similarly to anti-pattern A).

**Illustration: **One example of anti-pattern B is depicted on Figure 2 (i) where there are three copies of processor *Get_image_From_URL*, each copy receiving input data from distinct processors. The three copies are then merged into one single copy.

The next section will provide more details on how the transformations are extended to the entire workflow.

#### Safe Transformations

In this subsection, we introduce the notion of safe transformation. Intuitively, a transformation is safe if the semantics of the workflows is preserved (the outputs produced remain the same).

More formally, let *W*_1 _be a fragment of a workflow *W *consisting of r occurrences *P*^(1)^...*P*^(*r*) ^of a processor *P *such that there is no directed path between *P*^(*i*) ^and *P*^(*j*) ^(1 **≤ ***i ***≠ ***j ***≤ ***r*). Let *W*_2 _be a fragment of the workflow *W *consisting in one occurrence of *P *and possibly merge and split processors. A transformation that replaces *W*_1 _by *W*_2 _in the workflow *W *resulting in $W^{\prime }$ is safe if and only if: given the same workflow input values *In*, for any execution of *W *using *In*, named $\tilde{W}$, and any execution of $W^{\prime }$ using *In*, named ${\tilde{W}}^{\prime }$, the workflow output values *Out *obtained by $\tilde{W}$ and ${\tilde{W}}^{\prime }$ are the same.

It is straightforward to prove that the two transformations we propose to perform are safe.

### Refactoring approach

The previous section has introduced transformations able to locally remove anti-patterns. In this section, we will present the complete refactoring procedure we propose to follow. In particular, we have chosen not to remove all possible anti-patterns when such rewriting operations can make the transformed structures becoming more intricate than the original structures. Example of "simple" structures are *series-parallel *(SP) graphs [13] that are a specific kind of st-DAGs (directed acyclic graphs with one single source and one single target nodes) which provide well-known advantages in terms of complexity and ease-of-use in various situations (particularly when structures are to be compared [13]). SP-graphs have then naturally been used in the context of scientific workflows [14-16]. The challenge of our refactoring approach then lies in minimizing the presence of anti-patterns while ensuring that the number of structures which are not SP (called *non-SP structures*) will not increase. Note that it may be the case that our procedure transforms some non-SP structures into SP structures.

Without entering into the details, non-SP structures have some specific nodes called **reduction nodes **which cause the structure to be non-SP. Intuitively, a reduction node prevents from ranking the nodes of a DAG within series and parallel order. Details are provided in [17]. We will see how we apply our transformations to such nodes and we go back to this point in the Discussion section.

Additionally, in the following, we will also make use of the notion of **autonomous subgraph **introduced in the context of SP structures [17]. Intuitively, the autonomous subgraphs allow to restrict the initial graph to smaller components such that no edge comes in or goes out of the autonomous subgraph (except edges coming in the source of the autonomous subgraph or going out of its target). Several autonomous subgraphs can be nested. We will use this notion in order to apply transformations locally, without interaction with the rest of the graph.

#### Principle of the algorithm

The Refactoring algorithm takes in an st-DAG *G *and produces an st-DAG *DSG *from *G *by transforming the anti-patterns that can be removed from *G *while preserving its SP property. For it, the algorithm starts by identifying the set *SetAU *of autonomous subgraphs, and distills each of them, starting with the minimal ones, in a recursive way. Once each autonomous subgraph has been distilled, the whole graph *G *must be distilled in turn. Calls of the procedure *Distill *are done from a starting node *x *that can be either the source of an autonomous subgraph or a reduction node, or the source of *G*. We consider all the successors *p *of *x*, and search among all the other successors (and then descendants of *x*) whether there is a processor *q *that would be a copy of *p*. If it the case, we merge *p *and *q *according to the transformation for anti-patterns (A) and (B). Every time a transformation is performed, merging copies of a processor may give rise to new autonomous subgraphs, that lead to new distillations in turn. This last job is done by the procedure Down-Distillation.

**Figure 5:**
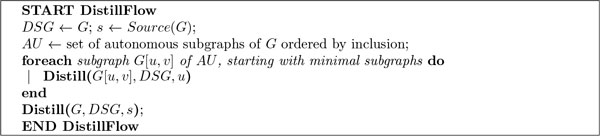
**Pseudo-code of the DistillFlow algorithm for removing anti-patterns**. Pseudo-code of DistillFlow, the algorithm that removes anti-patterns and provides a semantically-equivalent workflow. The additional file provides details on the Distill sub-procedure.

Figure 5 presents the main DistillFlow algorithm while the two procedures it uses (*DownDistillation *and *Distill*) for transforming workflows are available in the additional file, see Additional file 1. One major and additional function used by the procedure is introduced here after: *OKTransformation*(*p*, *q*, *GG*) which specifies the conditions for nodes *p *and *q *to be merged. It is true iff the following conditions are satisfied:

(i) *p *and *q *are copies of each other; (ii) *p *and *q *are involved in some anti-pattern (A) or (B) in *GG*; (iii) for any autonomous subgraph *G*' of *GG*, every time *p *appears in *G*', *q *appears in *G*' too. This last condition ensures us that we do not remove an anti-pattern by a transformation that would make an SP-graph becoming non-SP.

#### Illustration of the algorithm

**Figure 6:**
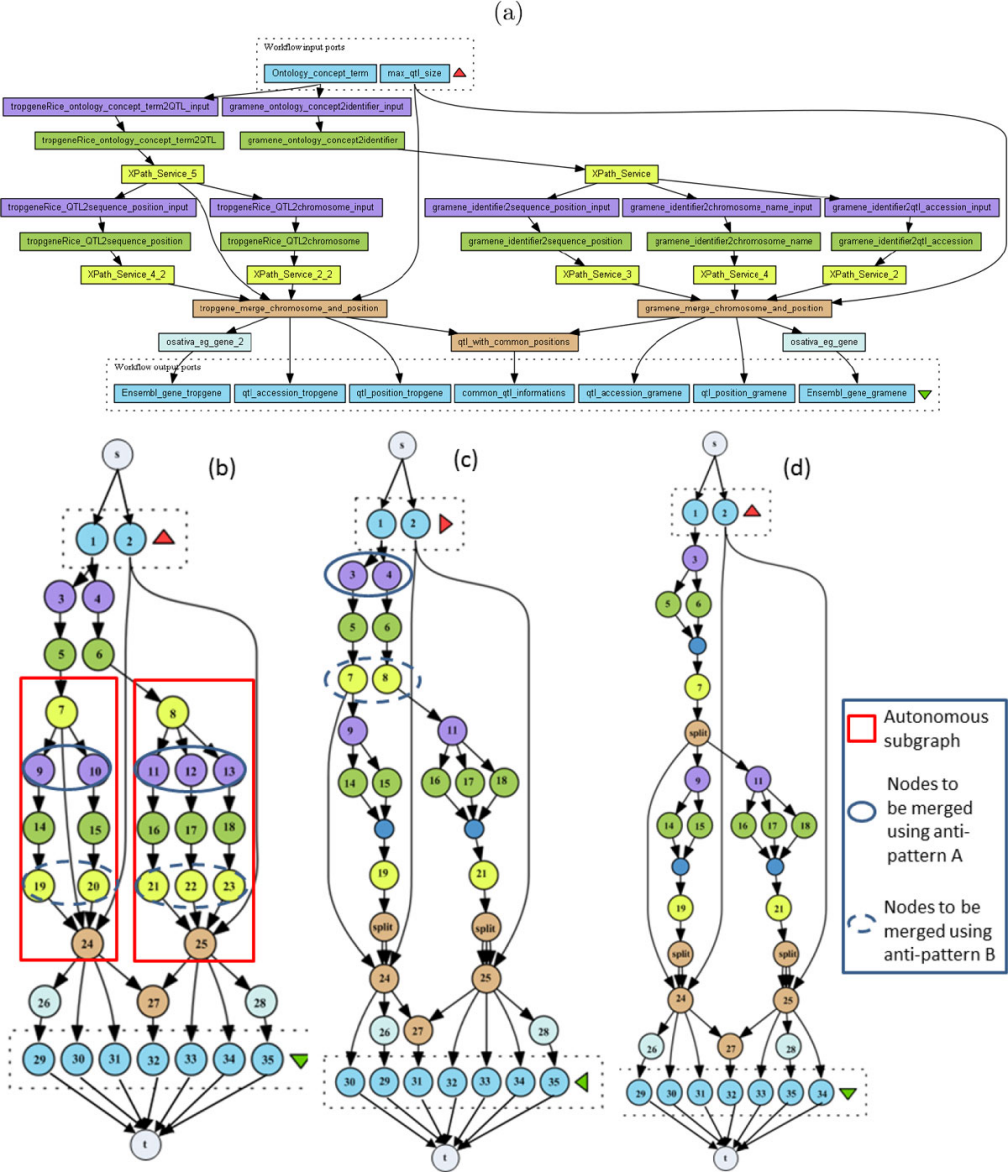
**Example of transformation**. Example of transformation of one workflow from myExperiment. (a) Original workflow; (b) Graph *G *representing the workflow; (c) Graph *DSG *obtained after distilling the two autonomous subgraphs; (d) Final distilled workflow obtained by Refactoring.

We propose to illustrate the execution of the **DistillFlow algorithm **on the workflow depicted in Figure 6 (a). We can see that it potentially contains several anti-patterns. Indeed, it duplicates processors many times: #3, #4, #9, #10, #11, #12, #13 all perform the same operation, and so do #7, #8, #19, #20, #21, #22, #23. The graph *G *representing the Taverna workflow is shown in Figure 6 (b). Note that this graph contains examples of autonomous subgraphs which are *G*[7,24], *G*[8,25] and *G*[3,24], where *G*[7,24] is nested in *G*[3,24].

At line 3 of the algorithm, autonomous subgraphs *G*[7,24] and *G*[8,25] are identified in *G*. At the first iteration of line 5, the procedure *Distill *is called with *G*[7,24] and node #7. During this recursive call, first nodes #9 and #10 are merged according to the transformation of anti-pattern (A), and then nodes #19 and #20, according to transformation of anti-pattern (B). At the second iteration of line 5, *Distill *is called with *G*[8,25] and node #8. During this recursive call, nodes #11, #12 and #13 are first merged (anti-pattern (A)), and then nodes #21, #22 and #23 (anti-pattern (B)). At line 7, *Distill *is called with *G*[*s*, *t*] and *s*. A first recursive call with *G*[2, *t*] and node #2 (successor of s that is a reduction node) does not change anything. Recursive calls starting with *G*[1, *t*] and node #1 (successor of *s *that is a reduction node) successively merge nodes #3 and #4 (anti-pattern (A)), and then nodes #7 and #8 (anti-pattern (B), Figure 6 (c)). Subsequent calls of *Distill *with *G*[24, *t*] and node #24, or with *G*[25, *t*] and node #25 do not imply any transformation. Note that nodes #9 and #11 are not merged since *OKTransformation*(9, 11, *GG*) is false (such a merge would have introduced a new reduction node, this point is discussed in the next section). Figure 6 (d) shows the final workflow where almost all the anti-patterns have been removed.

## Results

### Anti-patterns in workflow sets

We have applied the refactoring approach on two workflow sets: the public workflows from myExperiments and the private workflows of the BioVel project (http://www.biovel.eu), a consortium of fifteen partners from nine countries which aims at developing a virtual e-laboratory to facilitate research on biodiversity. BioVel promotes workflow sharing and aims at providing a library of workflows in the domain of biodiversity data analysis. Access to the repository to contributors, however, is restricted and controlled. Because of the restricted access and the focus on a specific domain of these workflows, they are broadly expected to be curated and thus of higher quality than the general myExperiment population.

For each workflow set, the total number of workflows, the number of workflows having at least one anti-pattern (of kind (A) or (B)) are provided in Table 1. Note that it is possible that the same workflow contains the two kinds of anti-pattern.

**Table 1:** Anti-patterns in workflow sets

Initial number of anti-patterns in workflow sets				
wf set	# wf	# wf **≥ **1 anti-pattern	# wf **≥ **1 anti-pattern (A)	# wf **≥ **1 anti-pattern (B)

myExperiment	1,454	374 (25.7%)	80 (5.5%)	359 (94.5%)

BioVel	71	29 (40.8%)	0	29 (100%)

Interestingly, 25.7% of the workflows of the myExperiment set contains at least one anti-pattern. Although anti-pattern A appears in only 5.5% of the total, it is particularly costly because it involves multiple executions of the same processor with the exact same input, therefore being able to remove it would be particularly beneficial. The prevalence of pattern B suggests that workflow designers may not know the list processing properties of Taverna (or functional languages).

As for the BioVel private workflows, 40.8% include at least one anti-pattern, all of kind B and thus none contains any kind A. Additionally, we have observed that a workflow from BioVel contains, on average, fewer anti-patterns than, on average, a workflow from myExperiment.

### Results obtained by DistillFlow

Table 2 provides the results obtained by DistillFlow in the two workflow sets: the number of workflows in which there is no remaining anti-patterns after applying the DistillFlow procedure, the number of workflows in which at least one anti-pattern has been removed.

**Table 2:** Results obtained by DistillFlow

Results obtained by DistillFlow in the two workflow sets		
wf set	# wf without any anti-pattern	# wf with at least one anti-pattern removed

myExperiment	302 (80.7%)	367 (98.1%)

BioVel	24 (82.7%)	29 (100%)

**Figure 7:**
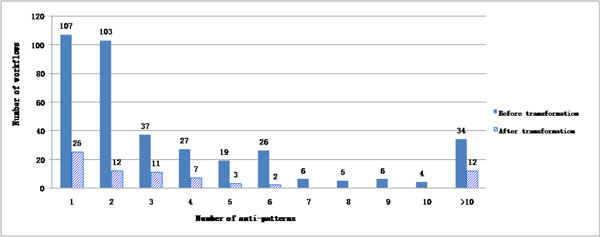
**Distribution of anti-patterns in myExperiment**. Distribution of number of anti-patterns among workflows in myExperiment, before and after applying DistillFlow.

In the set from myExperiment, DistillFlow is able to remove all the anti-patterns in 80.7% of the cases and at least one anti-pattern in 98% of the cases. 72 workflows are not completely free of anti-patterns after the DistillFlow process. However, the majority of these workflows has only one or two remaining patterns as indicated in Figure 7. More generally, Figure 7 shows that the number of remaining anti-patterns is low compared to the number of anti-patterns in original versions of workflows. Interestingly, additional experiments showed that on average three copies of processors are removed per workflow and this number is even particularly high for some workflows (up to 31).

**Figure 8:**
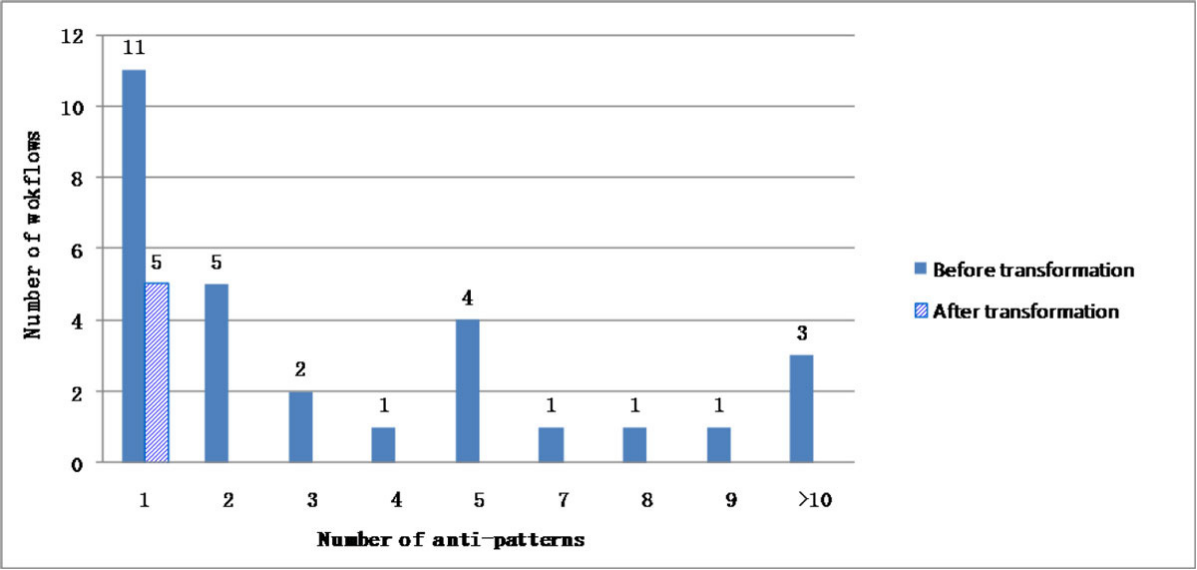
**Distribution of anti-patterns in BioVel**. Distribution of number of anti-patterns among workflows in BioVel, before and after applying DistillFlow (NB: no workflow in this set has 6 anti-patterns).

In the BioVel data set, DistillFlow is able to remove all the anti-patterns in 82.7% of the cases and at least one anti-pattern in all the workflows (100%). Only five (particularly big) workflows have remaining anti-patterns. All of them have actually one remaining anti-pattern, as indicated in Figure 8. Additional experiments allowed us to state that on this corpus, DistillFlow removes one node per workflow on average, compared to three in myExperiment. In very large workflows of BioVel (these are as large as the largest workflows in myExperiment), up to 15 nodes are removed, compared to 31 in myExperiment. In conclusion, the additional curation steps that occur in the BioVel community clearly make the produced workflows being of better quality; however some of these workflows could still benefit from our distilling approach.

## Discussion

### Simpler structures

**Figure 9:**
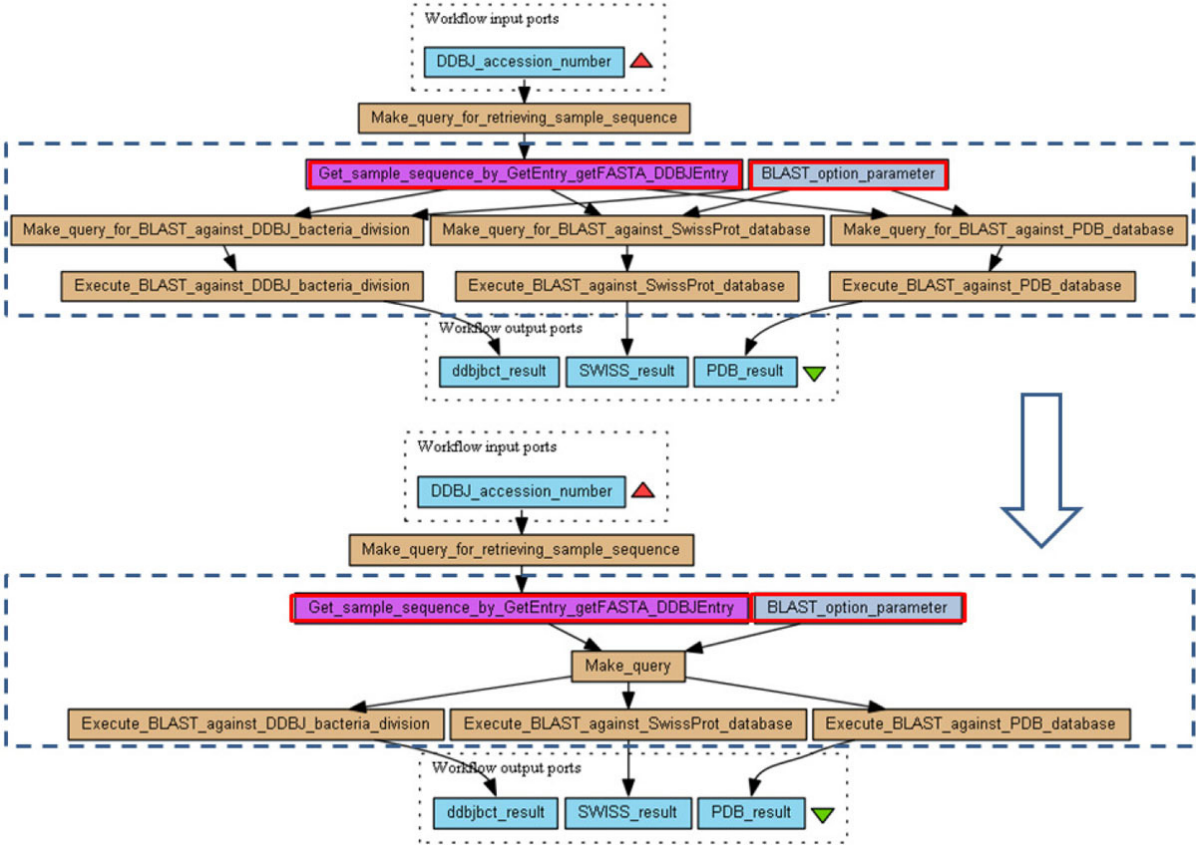
**Example of transformation using DistillFlow**. Example of transformation obtained using DistillFlow (original workflow at the top and rewritten workflow at the bottom).

**Figure 10:**
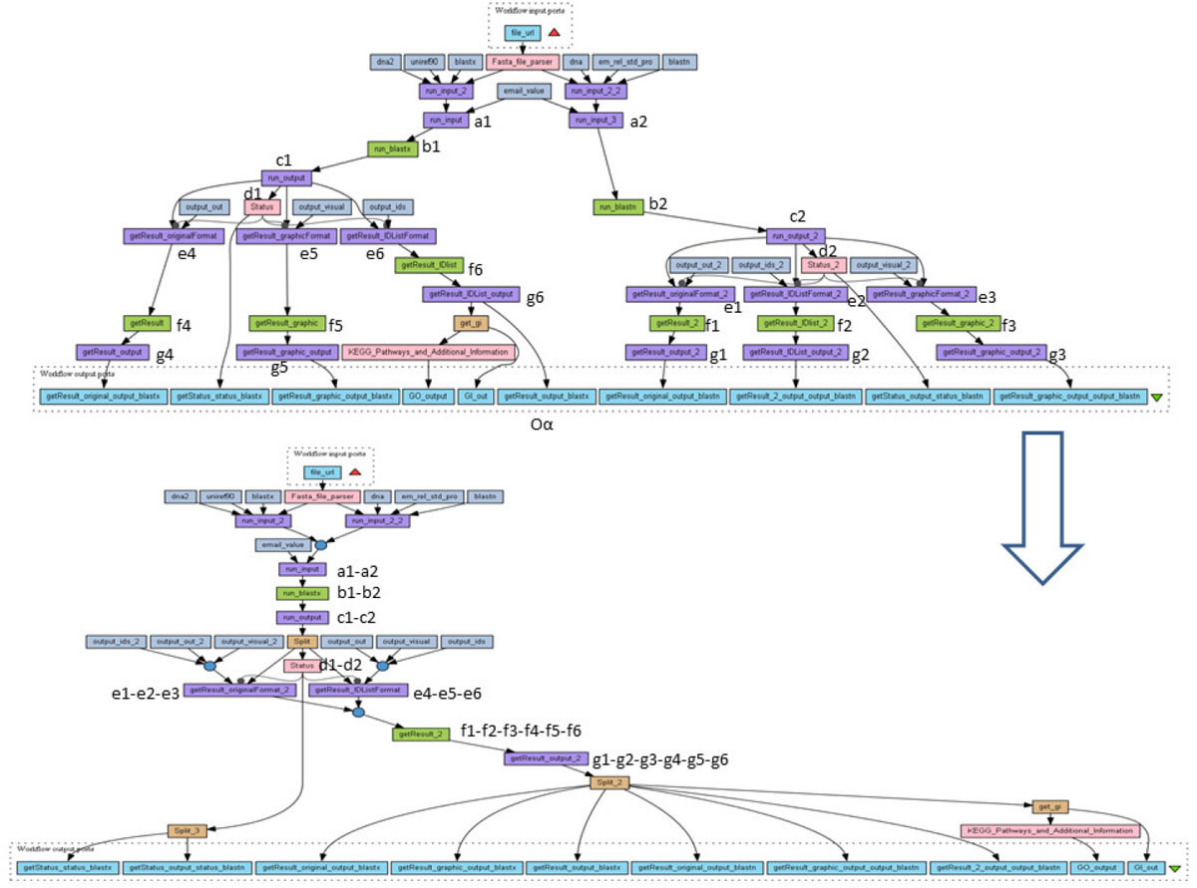
**Example of Non SP to SP transformation**. Example where the rewritten workflow becomes SP (original workflow at the top and rewritten workflow at the bottom).

When all the anti-patterns can be removed by DistillFlow, the resulting workflow structures are particularly simpler, as illustrated in examples provided all along the paper, including the two use cases (Figures 1, 2). Figures 9 and 10 provide two additional examples. In Figure 9, we have highlighted the rewritten subgraph that is particularly simpler compared to the same fragment of the workflow in the original setting. In Figure 10, the global structure is also simpler. Processors have been numbered so that the relationship between the two workflows (before and after the refactoring process) can be seen: in the original workflow *p_i _*denotes the *i^th ^*occurrence of processor *p *and in the rewritten workflow, *p_i _***− **... **− ***pj *denotes the node resulting of the merging of occurrences *p_i _***− **... **− ***pj*. For example, *f*_1_, *f*_2_, *f*_3_, *f*_4_, *f*_5_, *f*_6 _are all occurrences of the same processor which are replaced by one occurrence in the rewritten workflow (noted *f*_1 _**− ***f*_2 _**− ***f*_3 _**− ***f*_4 _**− ***f*_5 _**− ***f*_6 _in the rewritten workflow). As a result of the refactoring process on the workflow of Figure 10, three SPLIT processors have been introduced and 18 unnecessary duplications of processors have been removed.

### SP structures

As explained in the previous sections, DistillFlow acts carefully on the workflow structures, by removing anti-patterns (A) and (B) while never introducing new intricate structure as non-SP structure may be. Removing anti-patterns may actually automatically transform a non-SP structure into an SP structure as illustrated in Figure 9 in which the original workflow has two reduction nodes underlined in the figure (namely, *Get_sample_sequence_by GetEntry_getFASTA_DDBJEntry *and *BLAST_option_parameter*). While these nodes have several input/output links in the original setting they have (at most) one input link and one output link in the transformed version and they are not reduction nodes anymore.

More generally, in the myExperiment corpus, a total of 15 workflows had a non-SP structure before applying the refactoring algorithm and have an SP structure after.

However, it may also be the case that anti-patterns cannot be removed because removal would imply merging nodes which would create a new reduction node, making the structure of the transformed workflows more intricate. The number of reduction nodes is actually a commonly used metric to measure how far from an SP structure a structure may be [17]. In that sense, merging such nodes would make the rewritten workflow being further from an SP structure compared to the original workflow structure.

**Figure 11:**
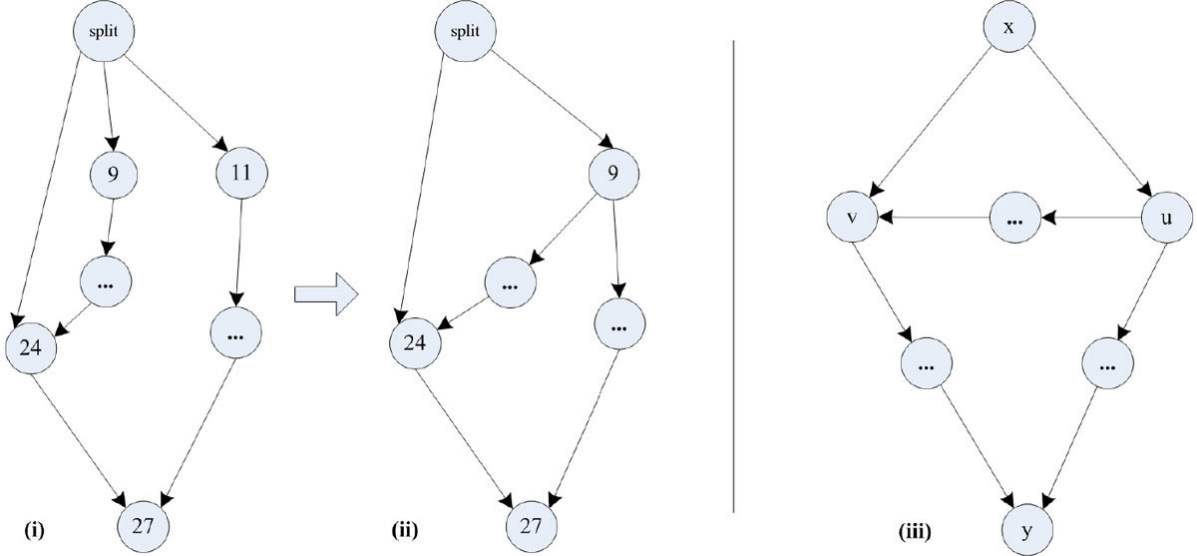
**More information on conditions for merging**. (i) Schematic view of a fragment of the workflow of Figure 6; (ii) Schematic view of the same fragment but nodes #9 and #11 are now merged; (iii) generic subgraph that is the cause of non-SP structure, where u is one reduction node.

65 workflows from the myExperiment corpus and five from the BioVel data set are involved in such a situation. The illustrative example for DistillFlow of Figure 6 is one such example: merging nodes #9 and #11 would introduce a new reduction node. In the original graph, node #9 appears in an autonomous subgraph while node #11 does not belong to this autonomous subgraph. If these two nodes were merged, the subgraph formed by all the paths from the SPLIT node to the node # 27 would have the structure of the subgraph responsible for non-SP structures (Figure 11 (iii)), and the merged node #9-11 would be the new reduction node. Figure 11 (i) shows a schematic view of a fragment of the original graph of Figure 6 while Figure 11 (ii) shows the structure obtained if nodes #9 and #11 were merged. It can be shown that in this graph ranking the nodes within series and parallel order is not possible anymore since the graph of Figure 11 (ii) is homeomorphic to the generic subgraph represented in Figure 11 (iii) which is the cause of non-SP structures [17].

A similar situation occurs in the workflow of Figure 10 in which nodes #e1-e2-e3 and #e4-e5-e6 cannot be merged by DistillFlow in order to avoid introducing one additional reduction node.

### Towards other kinds of (anti-)patterns

Another kind of situation that may occur is when the SP feature is not correlated at all with anti-patterns: the transformed workflows are free of anti-pattern but they still have non-SP structures.

A deep inspection of such workflows reveals that other kinds of patterns may be directly the cause of non-SP structures [18]. These patterns have a different nature from the anti-patterns considered so far in this paper in the sense that they cannot be removed while keeping the same workflow semantics. One of the most interesting pattern is probably the presence of intermediate processors which are directly linked to the workflow outputs. This situation occurs merely when users want to keep track of intermediate results and "forward" such results to the workflow outputs. We call such intermediate processors *trace nodes *and their outgoing edges linked to the workflow outputs are called *trace links*.

Several workflows depicted in this paper have *trace links*. For example, in Figure 10 on the top, the link that goes from the processor *g*_6 _directly to the workflow output *O_α _*is a trace link: when the workflow will be executed, the same data (produced by *g*_6_) will be sent both directly to the workflow output *O_α _*and to the downstream part of the workflow. By doing this, the workflow designer may want to keep track of the data produced by *g*_6_. However, as the processor *get*_*gi *will consume *O_α _*to produce to its turn some data, these produced data will have *O_α _*in their provenance information. *O_α _*will thus be automatically tracked by the provenance module of Taverna. The trace link from *g*_6 _to *O_α _*is then useless and could be removed. However, this removal should be done very carefully since removing trace links implies removing part of the workflow outputs. As a consequence, the signature of the workflow is changed which may have several consequences if the transformed workflow is used as a subworkflow within another bigger workflow that expects the subworkflow to provide given outputs. This kind of transformation should then be done in collaboration with the user so that s/he can estimate the impact of the changes.

## Conclusion

In this paper we have presented an algorithm, DistillFlow, which refactors Taverna workflows in a way that removes explicit redundancy making them possibly easier to use and share. DistillFlow is able to detect two kinds of *anti-patterns*, and rewrites them as new patterns which better exhibit desirable properties such as maintenance, reuse, and possibly efficiency of resource usage. This is achieved mainly by merging, under certain enabling conditions, multiple occurrences of the same workflow processors into one, while at the same time collecting the inputs to each of the original occurrences into a list. By virtue of Taverna's functional style of list processing, this refactoring can be proven to preserve the original workflow behavior.

We applied DistillFlow to two workflow collections, the one consisting of myExperiment public workflows, the other including private workflows from the BioVel project. Very interestingly, the number of anti-patterns per workflow and the number of duplicated nodes involved in each anti-pattern is also much lower in the BioVel workflow set than in the myExperiment workflow set. The additional curation and quality control effort that is placed on the BioVel collection, compared to the more heterogeneous workflows in myExperiment, is then confirmed by our study. We have shown that both data sets may still benefit from our approach.

## Related work

To the best of our knowledge, this is the first attempt at introducing a refactoring approach aiming at reducing workflow redundancy in the scientific workflows setting based on the study of workflow structure.

More research is available from the business workflows community, where several analysis techniques have proposed to discover control-flow errors in workflow designs (see [19] for references). More recent work in this community has even focused on data-flow verification [20]. However, this work is aimed primarily at detecting access concurrency problems in workflows using temporal logics, making both aims and approach different from ours. Also, it would be hard to transfer those results to the realm of scientific workflows, which are missing the complex control constructs of business workflows, and instead follow a dataflow model (a recent study [21] has shown that scientific workflows involve dataflow patterns that cannot be met in business workflows).

With the increase in popularity of workflow-based science, and bioinformatics in particular, the study of scientific workflow structures is becoming a timely research topic. Classification models have been developed to detect additional patterns in structure, usage and data [22]. More high-level patterns, associated to specific cases of use (data curation, analysis) have been identified in Taverna and Wings workflows [23]. Complementary to this work, graph-based approaches have been considered for automatically combining several analysis steps to help the workflow design process [24] while workflow summarization strategies have been developed to tackle workflow complexity [14,25].

## Future work

We intend to continue this work in several directions.

A first direction of research deals with generalizing our approach to other workflow systems. In particular, in systems able to exploit multi-core infrastructures or run on Grids or Cloud environments [26], our distilling approach could be highly beneficial. Indeed, as it pushes the management of multiple activations to system runtime, it can more efficiently parallelize their execution when deployed on a parallel architecture.

Another direction includes enriching the distilling approach with new patterns (such as *trace links*) and making it possible to choose whether or not such patterns should be transformed, in an interactive process. In such a framework, users might even have the choice to remove some anti-patterns even if the resulting workflow is non-SP, thus relaxing the SP-constraint. One of the challenges of such an approach will be to provide users with means to estimate the impact of their choices on the workflow structure and its future use.

Instead of considering an automatic procedure, the distilling procedure would be used during the design phase in a semi-automatic way. The refactoring approach would thus be built into the scientific workflow system design environment. It may then be complementary to approaches like [27] which help users find and connect tasks following an on-the-fly approach during the design phase or [28] which supports workflow design by offering an intuitive environment able to convert the users' interactions with data and Web Services into a more conventional workflow specification.

The longer term goal would then be to propose guidelines for workflow authors to more directly design *distilled *workflows. This work will be achieved in close collaboration with workflow authors and will involve conducting a complete user study to collect their feedback on the distilling approach and possibly resulting in finding again new anti-patterns.

## Abbreviations

DAG: Directed Acyclic Graph; SP: Series-Parallel; st-DAG: Directed Acyclic Graphs with one single source and one single target; wf: workflows.

## Competing interests

The authors declare that they have no competing interests.

## Author's contributions

JC has proposed a first list of anti-patterns and has designed the DistillFlow algorithm under the supervision of ChF and SCB. He has implemented DistillFlow and provided the results on the two sets of workflows. ChF and SCB have mostly written the paper. They have formalized and generalized the anti-patterns and the transformations. PM has designed and implemented the first concrete examples of workflows in Taverna involving transformations for anti-patterns; he has helped understanding some features of the functional model used in Taverna and significantly contributed to the writing of the paper. CG has contributed placing this work in the context of other refactoring approaches for scientific workflows. AR has provided very valuable information on several technical aspects of Taverna and helped understanding the results obtained in the BioVel workflows set. All authors have contributed to the writing of the paper.

## Supplementary Material

Additional file 1 **This document provides the complete pseudo-code of the DownDistillation and Distill procedures**. This file can be viewed with: Adobe Acrobat Reader (http://get.adobe.com/fr/reader/). Click here for file

## Declarations

Article processing charges for this paper have been paid from The University of Manchester's RCUK block grant.

This article has been published as part of *BMC Bioinformatics *Volume 15 Supplement 1, 2014: Integrated Bio-Search: Selected Works from the 12th International Workshop on Network Tools and Applications in Biology (NETTAB 2012). The full contents of the supplement are available online at http://www.biomedcentral.com/bmcbioinformatics/supplements/15/S1.
