# Supplementary material for: Distilling structure in Taverna scientific workflows: a refactoring approach
Source: BMC Bioinformatics. 2014 Jan 10;15(Suppl 1):S12. doi: 10.1186/1471-2105-15-S1-S12 (PMC4016501; doi:10.1186/1471-2105-15-S1-S12)
Supplement: Additional file 1 — This document provides the complete pseudo-code of the DownDistillation and Distill procedures. This file can be viewed with: Adobe Acrobat Reader (http://get.adobe.com/fr/reader/). [file 1471-2105-15-S1-S12-S1.pdf]

Distilling structure in Taverna scientific workflows: a refactoring approach – Additional file

Sarah Cohen-Boulakia*[1,2] , Jiuqiang Chen[1,2,3] , Paolo Missier[4] , Carole Goble[5] , Alan R Williams[5] and Christine Froidevaux[1,2]

[1]Laboratoire de Recherche en Informatique, CNRS UMR 8623, Université Paris Sud, France
[2]AMIB group, INRIA Saclay, France
[3]School of Information Science and Engineering, Lanzhou University, Lanzhou, Gansu, China
[4]University of Newcastle, United Kingdom
[5]University of Manchester, United Kingdom

Email: cohen@lri.fr; chenj@lri.fr; paolo.missier@ncl.ac.uk; carole.goble@manchester.ac.uk; alanrw@cs.man.ac.uk; christine.froidevaux@lri.fr;

*Corresponding author

Abstract

This document provides the additional file for the paper "Distilling structure in Taverna scientific workflows: A refactoring approach". It describes in details the algorithms designed in the refactoring approach introduced.

Pseudo code of the DownDistillation and Distill procedures

DownDistillation(IN $GG[q, v]$**, IN/OUT** $DSGG$**: graphs, IN** q**: node,**
IN/OUT $SetAU$**: set of graphs, IN/OUT** $ListRed$**: set of nodes)**
Distill($GG[q, v], DSGG, q$**)**;
$ListRed \leftarrow ListRed \cup$ {new reduction nodes of $GG[q, v]$};
$SetAU \leftarrow SetAU \cup$ {new autonomous subgraphs of $GG[q, v]$};
foreach *autonomous subgraph* $GG[a, b]$ *in* $SetAU$ **do**
  Distill($GG[a, b], DSGG, a$**)**
end
End DownDistillation

Figure 1: Pseudo-code of the DownDistillation procedure

Additional functions used are introduced here after.

- The following procedure makes use of the function **OKTransformation(p,q,GG)** which specifies the conditions for p and q to be merged. It is true iff the following conditions are satisfied:
  - p and q are copies of each other;
  - p and q are involved in some anti-pattern (A) or (B) in GG;
  - for any autonomous subgraph G' of GG, every time p appears in G', q appears in G' too. This last condition ensures us that we do not remove an anti-pattern by a transformation that would make an SP-graph becoming non-SP.
- The function **SameOrientedPath(p,q,GG)** is true iff there is at least a directed path dp in GG such that p and q belong to dp.
- **Visited** is a function allowing to mark nodes as visited or unvisited.

Note that, for example, in Figure 5 of the main paper, $OKTransformation(9, 11, GG)$ is false.

The next page provides the complete pseudo-code of the **Distill** procedure.

```
Distill(IN GG: graph; IN/OUT DSGG: graph; IN x: node)
v ← sink(GG);
ListRed ← set of reduction nodes of GG;
SetAU ← set of autonomous subgraphs of GG;
Visited(GG[q, v]) ← false /* set all the nodes of GG[q, v] unvisited */
foreach successor p of x in GG do
    /* search for copies of p */
    if Visited(p) ← false then
        p_1 ← p; flagp ← true;
        while flagp do
            /* flagp allows to consider all the unvisited descendant of p_1 if necessary */
            Distilled ← false /* Distilled says if some transformation on p_1 has been done */
            foreach successor q of x in GG, such that q ≠ p_1 do
                /* successors of x different from p_1 are potentially copies of p_1 */
                q_1 ← q; flagq ← true;
                while flagq do
                    /* flagq allows to consider all the unvisited descendant of q_1 if necessary */
                    if Visited(q_1)=false and SameOrientedPath(p_1,q_1, GG)=false then
                        if OKTransformation(p_1,q_1,GG)=true then
                            /* q_1 is a copy of p_1 in some anti-pattern and transformation can be performed */
                            transformation on DSGG, replacing q_1 by mergeq;
                            flagq ← false; distilled ← true; /* loop on q is stopped */
                        else
                            /* no transformation has been done on p_1 and q_1 */
                            if outDegree(q_1) ≠ 1 then
                                if there exists a single autonomous subgraph GG[q_1, y] in SetAU then
                                    q_1 ← y; /* the loop on q_1 is continued with the sink of the unique autonomous
                                        subgraph */
                                else
                                    /* there is no autonomous subgraph GG[q_1, y] in SetAU or more than one */
                                    if q_1 is a reduction node in Listred then
                                        /* search for anti-patterns from reduction node q_1 */
                                        DownDistillation(GG[q_1, v], DSGG, q_1, SetAU, ListRed);
                                        Visited(GG[q_1, v]) ← false;
                                        if outdegree(q_1) > 1 then flagq ← false;
                                    else
                                        flagq ← false;
                                        /* q_1 is not a reduction node or there is no autonomous subgraph GG[q_1, y] in
                                            SetAU the loop on q is stopped */
                                    end
                                end
                                q_1 ← the successor of q_1 /* outDegree(q_1) = 1 */
                            end
                        end
                        flagq ← false;
                    end
                end
            end
            /* while loop on flagp continues*/
            if distilled then
                /* if p_1 has been merged with some other node then search for anti-patterns from p_1 */
                DownDistillation(GG[p_1, v], DSGG, p_1, SetAU, ListRed);
                Visited(x, mergeq) ← true; /* set all the nodes on all paths from x to mergeq as visited */
            else
                /* p_1 has not been merged */
                if outDegree(p_1) ≠ 1 then
                    if there exists a single autonomous GG[p_1, y] in SetAU then
                        p_1 ← y;
                    else
                        flagp ← false;
                    end
                else
                    let p_1 ← the successor of p_1 /* outDegree(p_1) = 1 */
                end
            end
        end
    end
end
EndDistill
```

Figure 2: Pseudo-code of the Distill procedure
